# Neutrophil-Dependent Immunity During Pulmonary Infections and Inflammations

**DOI:** 10.3389/fimmu.2021.689866

**Published:** 2021-10-19

**Authors:** Clement Yaw Effah, Emmanuel Kwateng Drokow, Clement Agboyibor, Lihua Ding, Sitian He, Shaohua Liu, Senyo Yao Akorli, Emmanuel Nuamah, Tongwen Sun, Xiaolei Zhou, Hong Liu, Zhiwei Xu, Feifei Feng, Yongjun Wu, Xiaoju Zhang

**Affiliations:** ^1^ College of Public Health, Zhengzhou University, Zhengzhou, China; ^2^ Department of Radiation Oncology, Zhengzhou University People’s Hospital & Henan Provincial People’s Hospital, Zhengzhou, China; ^3^ School of Pharmaceutical Sciences, Zhengzhou University, Zhengzhou, China; ^4^ General ICU, Henan Key Laboratory of Critical Care Medicine, The First Affiliated Hospital of Zhengzhou University, Zhengzhou, China; ^5^ College of Agriculture and Natural Sciences, University of Cape Coast, Cape Coast, Ghana; ^6^ Department of Respiratory, Henan Provincial Chest Hospital, Zhengzhou, China; ^7^ Department of Respiratory, The First Affiliated Hospital of Zhengzhou University, Zhengzhou, China; ^8^ Department of Respiratory and Critical Care Medicine, People’s Hospital of Zhengzhou University & Henan Provincial People’s Hospital, Zhengzhou, China

**Keywords:** lung, innate immune response, neutrophils, chemokines, pattern recognition receptors, biomarkers

## Abstract

Rapid recruitment of neutrophils to an inflamed site is one of the hallmarks of an effective host defense mechanism. The main pathway through which this happens is by the innate immune response. Neutrophils, which play an important part in innate immune defense, migrate into lungs through the modulation actions of chemokines to execute a variety of pro-inflammatory functions. Despite the importance of chemokines in host immunity, little has been discussed on their roles in host immunity. A holistic understanding of neutrophil recruitment, pattern recognition pathways, the roles of chemokines and the pathophysiological roles of neutrophils in host immunity may allow for new approaches in the treatment of infectious and inflammatory disease of the lung. Herein, this review aims at highlighting some of the developments in lung neutrophil-immunity by focusing on the functions and roles of CXC/CC chemokines and pattern recognition receptors in neutrophil immunity during pulmonary inflammations. The pathophysiological roles of neutrophils in COVID-19 and thromboembolism have also been summarized. We finally summarized various neutrophil biomarkers that can be utilized as prognostic molecules in pulmonary inflammations and discussed various neutrophil-targeted therapies for neutrophil-driven pulmonary inflammatory diseases.

## Introduction

Infections of the lower respiratory tract accounts for about 35% of all deaths accruing from infectious diseases, resulting in a yearly death rate of about 4 million patients (1). They increase the worldwide disease burden than most infectious diseases such as HIV infection and malaria (1, 2). The effectiveness of a host defense mechanism against infections of the lung is very crucial and this is basically dependent on the quick clearance of the disease-causing agent from the airways. The main pathway through which this happens is by the innate immune response (3, 4). Therefore, any inabilities in the host innate immune response can lead to heavy microbial colonization which can compromise the integrity of the lung parenchyma. The exact mechanism of immune activation during pulmonary inflammation and infection remains unclear. However, some studies have iterated that the host, through resident and non-resident immune cells and receptors such as pattern recognition receptors (PRR), recognizes and destroys these organisms. One of the key players in innate immunity is neutrophils and their recruitment to the site of inflammation. Because of their ability to enter into organs and tissues to execute important host defense mechanisms, neutrophils are usually refer to as all-terrain vehicle of the innate immune system. Neutrophils form the first line of host defense mechanism because of how quick they are recruited when the lung is challenged with a microbial infection or other particles. These functions of neutrophils are highly regulated by signals received from their repertoire of PRRs, and this allow neutrophils, whose recruitment are modulated by chemokines to sense both pattern-associated molecular patterns (PAMPs) and damage-associated molecular patterns (DAMPs) at the site of inflammation. Robust recruitment of neutrophils to an inflamed site is the hallmark of all injuries and acute microbial infections (5, 6). This robust recruitment is made possible through neutrophil concentration gradients across the epithelium, extracellular matrix (ECM) and endothelium (7, 8). Neutrophils are guided to the sites of action by chemokines expressed by resident cells. It has been confirmed through animal and clinical studies that, CXC and CC chemokines play important roles in innate immunity by recruiting and activating neutrophils during microbial infections and injuries of the lungs. During some inflammatory diseases, the levels of these chemokines increase and vary according to the stage of the disease (9–11). Inappropriate neutrophil recruitment, or when neutrophil activation is impaired, it can lead to lung inflammations. Also, when the recruitment is not controlled or when the activation is not sustained, this could lead to collateral tissue damage and disease.

Despite the importance of chemokines and pattern recognition receptors in host immunity, little has been discussed on their roles in neutrophil-induced immunity. Herein, this review aims at highlighting some of the developments in lung neutrophil immunity and focuses on the functions of (1) CXC chemokines, (2) CC chemokines, (3) pattern recognition receptors and (4) integrins in neutrophil immunity during lung infections and inflammations. The pathophysiological role of neutrophils in COVID-19 and thromboembolism have also been summarized. Finally, we discussed various neutrophilic biomarkers, and various neutrophil-targeted therapies for neutrophil-driven pulmonary inflammatory diseases.

## Neutrophils

**Figure 1:**
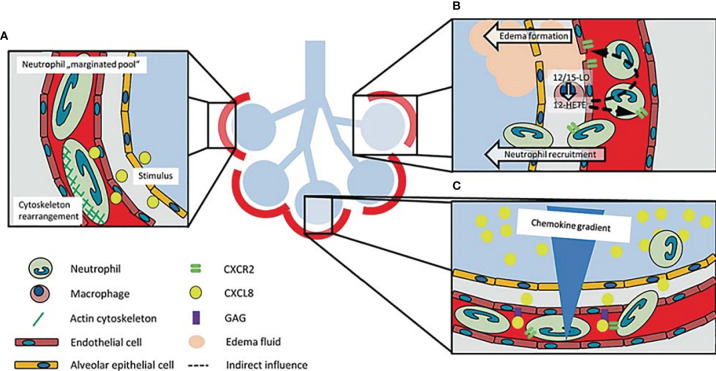
Schematic representation of how neutrophils are recruited to the lung. **(A)** Neutrophils change their shapes in order to move across the capillary bed because of the small nature of the lung capillaries. The increase in time required by neutrophils to transit in the lungs accounts for the name ‘marginated pool of neutrophils’. Neutrophil stiffening by cytoskeleton rearrangement after stimulation participates in neutrophil recruitment into the lung. **(B)** 12-HETE, a lipid mediator made by 12/15-LO in lung macrophages regulates the balance of chemokine-chemokine receptors and increases vascular permeability and neutrophil recruitment in the lungs. **(C)** The increase in chemokine concentration gradient in the alveolar region coupled with the presentation of chemokines by glycosaminoglycans, modulates the recruitment of neutrophils to this region of the lung (18).

In humans, neutrophils form the largest proportion of leukocytes that circulate in the blood and form the major component in organs such as the lungs. They play a major role in innate immunity despite being described as having terminal differentiation and being characterized with a short lifespan after leaving the hematopoietic organ. Their main distinguishing characteristic is the removal of debris and pathogens through phagocytosis but can also play important roles in immune functions. Aside the direct phagocytosis of bacteria (12) and fungi (13), neutrophils, through the process of NETosis reduce the spread of microbes by releasing neutrophil extracellular traps (NETs) (14). Although neutrophils destroy pathogenic agents, they also have the ability to significantly modulate the functions of other immune cells. The recruitment of neutrophils into the lung usually occurs in the small capillaries of the alveolar network (15, 16). Neutrophils change their shapes in order to move across the capillary bed because of the small nature of the lung capillaries (15). In addition, the velocity of blood flow within the lungs’ capillary network is low (17). The velocity of blood flow and the change in shape of neutrophils account for the increase in the time required by neutrophils to transit during physiological conditions in the pulmonary microvasculature and this has accounted for the name ‘marginated pool of neutrophils’ (15) (**Figure 1A**). The extracellular secretion of oxidases and proteases (e.g. Myeloperoxidase and neutrophil elastase) following the mobilization of granule to the surface of a cell is a trademark of neutrophil activities within an inflamed airway. This mechanism modulates the upregulation of primary and secondary granule markers (CD63 and CD66b, respectively) on the surface of airway neutrophils resulting in high proteolytic and oxidative activities. Further findings have shown that airway neutrophils play a role in the regulation of adaptive immune system, demonstrating the multifaceted significance of neutrophil plasticity (19). For instance, in cystic fibrosis airway neutrophils, a significant immunosuppressive feature has been detected. This feature is immunosuppressive because it causes the downregulation of T-cell through the activation of arginase I (20). When neutrophils are activated in cystic fibrosis airways, they don’t only impact on T cells but also play important role in the lymphatic compartment by showcasing its antigen-presenting cell capabilities (e.g., expression of CD80, CD86, and MHC II). One feature of immature neutrophils is the expression of CXCR4 and is highly upregulated when airway neutrophils are activated. This could be responsible for their acquired ability to migrate from inflammatory tissues into lymphatic vessels (21–23). The transiting of neutrophils to lymph nodes has been related to the proliferation of T-cell, suggesting that neutrophils are also involved in the active regulation of the adaptive immune response (23, 24). Intracellular pathogens can use this lymphatic neutrophils as a “Trojan horse” to spread within the body (25, 26).

## Integrin-Mediated Neutrophil Interaction With Endothelia and Epithelial Cells

Until recently, adhesion of neutrophils to the endothelium was known to involve three phases: rolling which is mediated by selectin, chemokine-triggered activation of neutrophils, and firm arrest of neutrophils initiated by integrins (27, 28). However, studies have shown that this process is a rather complex event made up of additional phases such as tethering, slow rolling, modulation of adhesion strength, intraluminal crawling, and transcellular or paracellular migration (27, 29). Currently, it is believed that the first stage in neutrophil and other leukocyte adhesion to the endothelium is neutrophil capture (or tethering), which is mediated by interactions between L, E, and P-selectins, as well as P-selectin glycoprotein ligand (PSGL1) and α4β1 (VLA4) integrin. Leukocytes, inflamed endothelium and platelets, and inflamed endothelium are known to respectively express L-selectin, P-selectin and E-selectin. Also, endothelium and some leukocytes are known to express PSGL1. Subsequently, an interaction between selectins, PSGL-1 and other glycosylated ligands mediate the rolling of neutrophils on the endothelium. More importantly, adhesions which are mediated by L-selectin and P-selectin require shear stress (30, 31). This phase is ensued by a selectin-triggered signaling phase (“slow rolling”), followed by a firm capture of neutrophils on the endothelial surface, a step that involves β1- and β2-integrins and their respective binding partners.

Integrins are αβ heterodimeric, transmembrane proteins that mediate both cell-substrate and cell-cell interactions with a diverse group of ligands (32). Integrins have numerous functions, one of which is to mediate cell migration. Integrins are made up of several α subfamily (α2β1, α3β1, α4β1, α5β1, α6β1 and α9β1) which are usually expressed and upregulated on neutrophils (33, 34). Integrins, including those of the β1 and β2 subfamilies are known to be expressed by human neutrophils. Proteins of the extracellular matrix (ECM) binds to the β1 family integrin upon recognition of the amino acid sequences Arg-Gly-Asp (RGD) and Leu-Asp-Val (LDV) (35). The β2-integrins consist of a common β-chain (CD18) and a variable α-chain (CD11a, b, c, or d). Interactions between CD11a/CD18 (LFA-1), α4β1 (VLA-4), α4β7 and some intercellular adhesion molecules such as ICAM-1, VCAM1, and MADCAM1 mediate neutrophil arrest. A series of outside-in and inside-out intracellular signaling pathways are then activated, resulting in the strengthening of adhesion followed by the spreading of neutrophils. Conformational changes in the structure of inserted (I) domain of the αL subunit of LFA-149 enhance firm neutrophils adhesion under shear flow (36, 37). Neutrophils then crawl along endothelial cells (“intraluminal crawling”) by a process involving interactions between CD11b/CD18 (αmβ2 or Mac-1) and ICAM-1. Neutrophils, through a paracellular or a transcellular route, finally transmigrate across the endothelium. In a murine experiment, integrin α4β1 was found to be involved in neutrophil adhesion during pneumonia caused by *Streptococcus pneumoniae* (38). Also, in a study by Ulyanova et al., it was opined that integrin α4β1 plays a key role in adhesion and migration during lung inflammation, and mediates integrin β2-independent neutrophil accumulation (39). However, the expression of integrin α4β1 on neutrophils in early stage acute respiratory distress syndrome (ARDS), was found to be downregulated (40). During lung infection, it has been reported that extravasation of neutrophils to the site of inflammation is aided by integrin α9β1 (33). In a study involving older people with aspiration pneumonia, it was found that integrin α9β1 and CD11b expression levels on circulating neutrophils were increased (41).

## Neutrophil Degranulation

Pro- and anti-inflammatory substances are the major components of neutrophil granules and these can be released to destroy pathogenic organisms. This process is termed as degranulation (42). Neutrophils play important roles by releasing granules that aid in the killing of invading pathogens. Primary (azurophilic), secondary (specific) and tertiary (gelatinase) granules, as well as secretory vesicles are the four types of granules found in neutrophils. Each of these granules has a different protein content (43). They have distinct functions and are released sequentially to cell surface or to the microbe-containing phagolysosome by exocytosis in response to various signals. Following initial contact of neutrophils and endothelial cells, secretory vesicles are released *via* exocytosis which results in the expression of some key surface membrane proteins leading to the rolling of neutrophils through the endothelial monolayer in blood vessels. This then initiates extravasation at the infection site (43). The release of a secretory vesicles is respectively followed by the release of tertiary, primary and secondary granules (16). The release of the contents of the primary and secondary granules into the phagolysosome, or degranulation into surrounding tissues, initiates a series of antimicrobial activities. Antimicrobial enzymes and peptides such as serine proteases and defensins, as well as myeloperoxidase, which converts H_2_O_2_ to antiseptics hypobromous acid, hypochlorous acid and hypoiodous acid are all constituents of the primary granules (44). Lactoferrin, lipocalin, lysozyme, LL37 and matrix metalloproteinases are among the overlapping proteins contained in secondary and tertiary granules (44). The actions of these molecules within the granules are required for effective pulmonary immunity without causing significant tissue damage (45).

## Neutrophil Extracellular Traps

**Figure 2:**
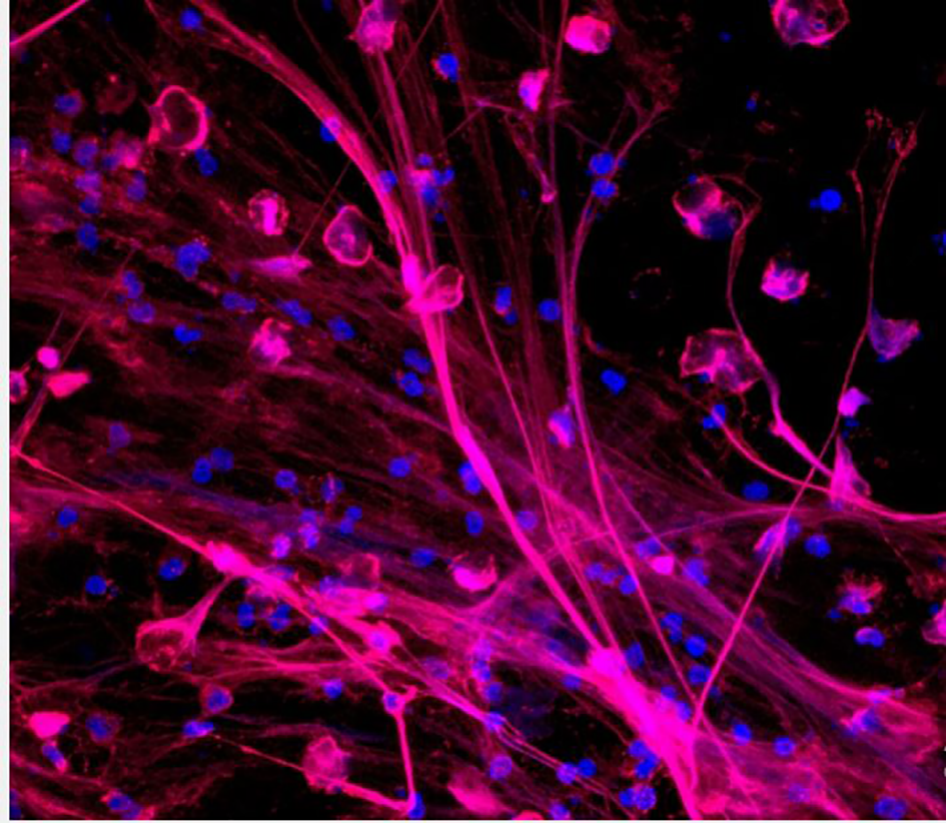
Neutrophils releasing neutrophil extracellular traps (NETs). NETs are stained to visualize neutrophil myeloperoxidase (red) and DNA (blue) (46).

Until recently, neutrophils were mainly known to utilize both extracellular killing by exocytosis and intracellular killing by phagocytosis to recognize and kill pathogenic organisms. Recently, the release of extracellular “traps” or complexes created by cationic effectors (including neutrophil myeloperoxidase and elastase), histones, and decondensed nuclear DNA (e.g., after histone citrullination), have been identified as the third effector mechanism utilized by neutrophils. The release of neutrophil extracellular traps (NETs) (**Figure 2**), through a process call “NETosis”, is believed to immobilize pathogens and probably destroy them while also precipitating neutrophil death (47, 48). Although NETs have been linked to a variety of diseases, including viral, fungal, and bacterial infectious diseases as well as other autoimmune disorders, their functions in chronic and acute inflammation is still not fully elucidated (47, 49). Studies focusing on NETs have demonstrated both advantageous and harmful impacts of these systems in the context of airway diseases (50). NET can increase the killing efficiency and reduce the burden caused by pathogens because of its ability to spread out and trap these pathogens. A lot of organisms have developed a mechanism to evade these destructive impacts of the NET system and this can result in the accumulation of host DNA, histones, neutrophil elastase, and myeloperoxidase thereby causing direct or indirect cell toxicity and subsequent lung injury (51–53). This can also lead to obstruction in the airways because the presence of these proteins and enzymes may result in an increased mucus viscosity (54–56).

## Regulatory Activities of Neutrophils During Pulmonary Inflammation and Infection

The most common lung infectious disease which is characterized by inflammation in the interstitial lung, alveolar and the airways is pneumonia. Pneumonia development hinges on the interplay between mucosal immunity and mucosal colonization by the etiological agent (57). During pneumonia, there is recruitment of neutrophils into the lung. Neutrophils can act in unison with other immune cells to regulate infections caused by pathogens (58). In recent times, evidence have been documented on the roles of neutrophils in pneumonia pathogenesis. According to Onishi et al. (59), the level of neutrophil in bronchoalveolar lavage fluid (BALF) was greater in the relapse category of organizing pneumonia patients. On the other hand, community-acquired pneumonia patients had lower levels of neutrophils in peripheral blood (60). During acute lung injury (ALI), chemokines present on the inflamed pulmonary endothelial cells play important roles in the recruitment of neutrophils into the lung (61). Intracellular signaling cascade is initiated through the binding of chemokines to their neutrophil receptors which leads to integrin activation and cytoskeleton rearrangement. This process is essential for the recruitment of neutrophils (61). As a response to lung injury, resident pulmonary macrophages produce and transmit chemokines, and also server as a major source of pro-inflammatory mediators, such as IL-1, tumor necrosis factor- (TNF-α), 12-hydroxyeicosatetraenoic acid (12-HETE), and interleukin (IL)-8 (CXCL8) (62, 63). During inflammation, the lung macrophages/monocytes express 12/15-lipoxygenase (12/15-LO). The 12-HETE lipid mediator, a product of 12/15-LO, has been involved in the regulation of vascular permeability and recruitment of neutrophils into the lungs during lipopolysaccharide-induced injury in the lungs (**Figure 1B**) (63, 64). The 12/15-LO is known to be essential for the mobilization of neutrophil into the lung’s intra-alveolar and interstitial regions by hematopoietic cells. However, trafficking of neutrophils to the lung’s microvasculature is controlled by non-hematopoietic 12/15-LO (64). This was confirmed when the vascular permeability was drastically reduced in both 12/15-LO-deficient and 12/15-LO-blocked WT mice during an ALI-induced experiment. A CXCR2-dependent mechanism has also been shown to mediate vascular permeability (**Figure 1B**) (63, 64). A concentration gradient of chemokine within the intravascular, interstitial and the alveolar space has been observed during the development of ALI, with the maximum accumulation been observed in the alveolar area (**Figure 1C**) (65, 66). The migration of neutrophils into the alveolar area is stimulated by this gradient. Distinct chemokines presented by glycosaminoglycans get stuck to endothelial cells (67, 68). CXCL8’s monomer-dimer equilibrium is vital for the attachment of CXCL8 to glycosaminoglycans which influences its capacity to mobilize neutrophils (69). CXCR2 receptors on neutrophils have been found to be among the key significant chemokine receptors which are active in lung neutrophil mobilization during ALI animal models (**Figure 1C**) (70–73).

One of the hallmark of acute respiratory distress syndrome (ARDS) is the infiltration of neutrophils to the inflamed lung (74). Endothelial cells stimulate and arrest circulating neutrophils in patients with ARDS (75). The formation of neutrophil extracellular traps (NETs), oxidative stress and the release of proteases usually occurs when there is activation of neutrophil during ARDS. During ARDS, selectin sequesters neutrophils, leading to an “inside-out” activation of CD11a/CD18, which then binds to intercellular adhesion molecules (ICAMs) of the endothelium (76). During ARDS, neutrophils help to repair the damaged lung tissue by releasing MMP-9 and activating the Wnt/b-catenin pathway (77). The role of neutrophil recruitment in ARDS is complicated, and more research is required. Some researchers have shed light on the pathogenic function of neutrophils in inflammatory ARDS (78). Higher levels of neutrophils have been observed in patients presenting with ARDS and this can serve as a predictor of poor prognostic outcome (79). As a result, it has been projected that strategies to reduce neutrophils in lung tissue, including the reduction of neutrophil recruitment and the activation of its immune functions, would reduce lung injury. Elevated levels of neutrophils in pulmonary tissues contribute to the pathogenesis of ARDS, while decreasing levels reduce the generation of cytotoxic mediators (80). The assembly and activation of reactive oxygen species (ROS)-producing nicotinamide adenine dinucleotide phosphate (NADPH) oxidase complex (NOX2) by neutrophils can contribute to the progression of ARDS (81). NOX2, which is found on membranes, converts oxygen to superoxide anion, which is then released to the outside of the cells. The highly reactive superoxide anion spontaneously dismutates into a more stable hydrogen peroxide (H_2_O_2_). H_2_O_2_ can pass through the cell membrane and disseminate to the extracellular or intracellular environment. H_2_O_2_ is used by the enzyme myeloperoxidase to generate hydroxyl radicals, hypochlorous acid, and other reactive products (82). Since ROS are harmful to pulmonary tissues, it is desirable to reduce ROS production in order to reduce lung inflammatory injury (83).

## Pattern Recognition Receptors in Pulmonary Infections and Inflammation

**Figure 3:**
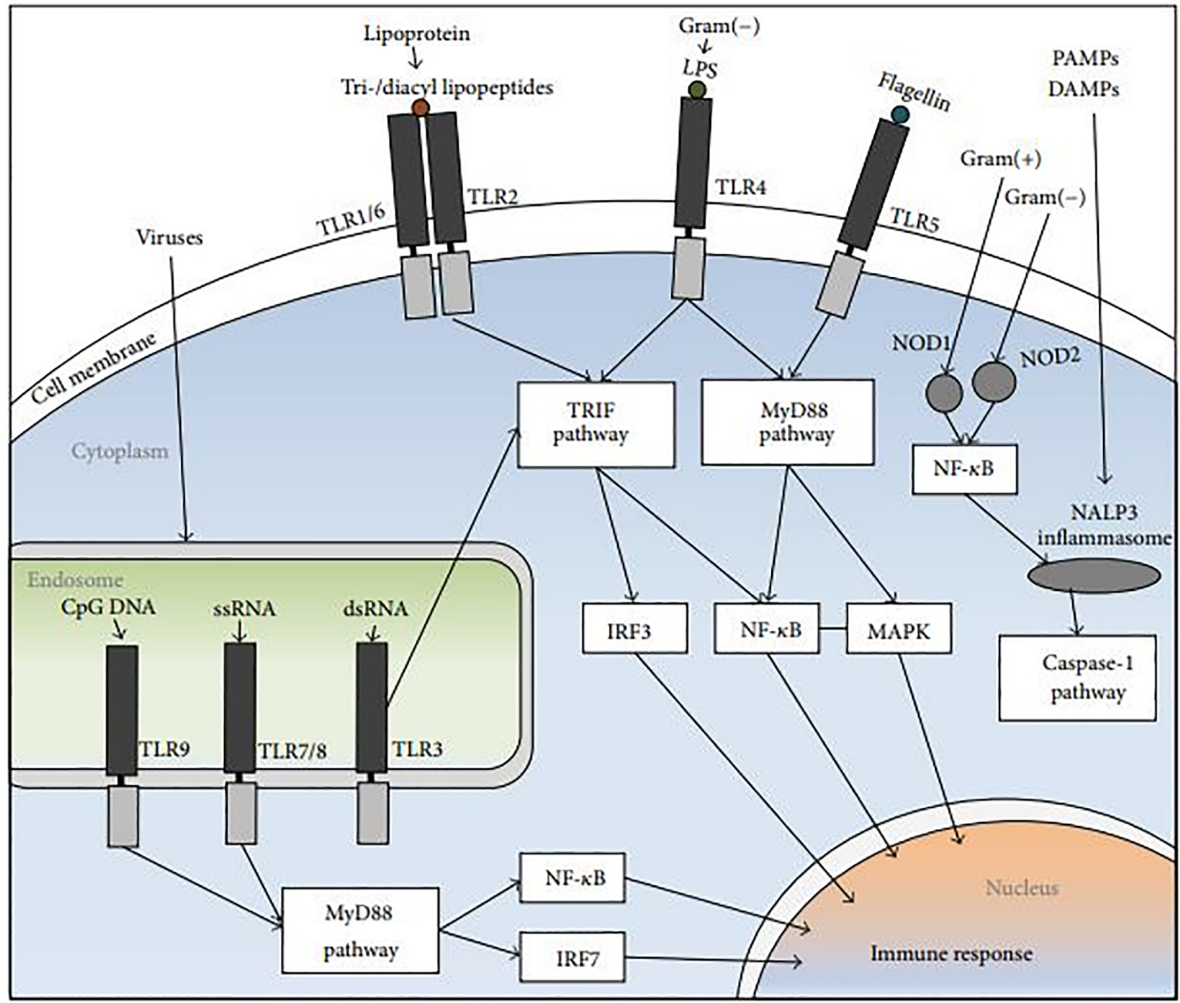
The NLR and TLR signal-transduction pathways. The PRRs identify DAMPs and PAMPs. Endosomal TLR3, and TLR4, TLR1/6+TLR2 heterodimers stimulate the TRIF pathway, followed by NF-κB and IRF induction. The MyD88 pathway is activated by TLR5 and endosomal TLR9 and TLR7, followed by IRF7, NF-κB and MAPK activation. NOD2 and NOD1 which are cytoplasmic PRRs, induce the recruitment of NALP3 inflammasome, trigger the release of NF-κB, and activate the caspase-1 pathway (108).

The identification of pathogens during infection is one of the most significant roles of the innate immune system and this recognition is driven by cell-surface pattern recognition receptors (PRRs) (84). Intracellular and extracellular PRRs ligation can mediate chemokine/cytokine expression and also induce neutrophil recruitment into the lungs during lung inflammations. PRRs specifically recognize unique molecular patterns found on the surfaces of microbes and this leads to a series of upstream and downstream events. This eventually causes the migration of neutrophils to the lungs followed by the influx of monocyte/macrophage to the site of infection (57). The first host cells that encounter antigens of microorganisms during infection are the airway epithelial cells, dendritic cells, and alveolar macrophages. These cells trigger proinflammatory or anti-inflammatory downstream immune responses. PRRs are present in soluble forms like mannan-binding lectin (MBL) and in the form of transmembraneous or intracellular molecules that directly mediate cellular immune responses. The major families of airway epithelial PPRs include protease-activated receptors (PAR), Toll-like receptors (TLRs), Nod-like receptors (NLRs), C-type lectin receptors, RIG-I-like receptors (RLRs), and the bitter- and sweet-taste receptors (**Table 1**). These PRRs initiate a cascade of downstream signal transduction pathways which leads to the recognition of PAMPs and DAMPs in response to pulmonary infections (**Figure 3**). PAMPs are highly conserved structures presented by several groups of microorganisms. PAMPs may include bacterial lipopolysaccharide (LPS), peptidoglycan (PGN) or lipoteichoic acid (LTA).

**Table 1:** Summary of some pattern recognition receptors and their functions in innate immunity.

Ligand (adaptors in parentheses)	PRRs	Ligand (origin in parentheses)	Localization	Function in neutrophil	Signals	Response	Ref
**TLRs** (TRAM, Trif, Mal, MyD88)	TLR1	Triacyl lipopeptides (bacterial lipoprotein) Di-/triacyl lipopeptides	Cell surface	Migration, Inhibit apoptosis, NET formation, Respiratory burst, Degranulation, Phagocytosis	IRFs NF-κB MAPKs	Pro–IL-1, pro–IL-18 Antiviral proteins Chemokines Cytokines	(85, 86)
	TLR2	Multiple lipoproteins, Lipoteichoic acid, Zymosan (fungi)	Cell surface	Formation of heterophilic dimers with TLR1 and TLR6, Respiratory burst, Neutrophil migration, Degranulation, apoptotic regulator, Phagocytosis			(87, 88)
	TLR4	LPS (Gram-negative bacteria)	Cell surface	Recognition of LPS together with myeloid differentiation factor 2, Respiratory burst, NET formation, Neutrophil migration, Phagocytosis, Inhibit apoptosis, Degranulation			(89–91)
	TLR5	Flagellin (flagellated bacteria)	Cell surface	Activation of lung epithelial cells to induce inflammatory cytokine, NET formation, Inhibit apoptosis, Phagocytosis, Degranulation			(92, 93)
	TLR6	Triacyl lipopeptides (bacterial lipoprotein)	Cell surface	Formation of heterophilic dimers with TLR2, Respiratory burst, Inhibition of apoptosis, Phagocytosis, NET formation, Degranulation,			(94)
	TLR8	ssRNA (viruses); small antiviral compounds	Endosome	Recognition of synthetic compound imidazoquinoline, Neutrophil migration, NET formation, Respiratory burst, Degranulation, Inhibition of apoptosis, Phagocytosis			(90, 91)
	TLR9	Unmethylated CpG DNA	Endosome	Degranulation, Phagocytosis, Respiratory burst, Inhibit apoptosis, NET formation, Migration, Proinflammatory cytokines			(95, 96)
	TLR10	Unknown		No neutrophil function described			(94, 97)
**NLRs** (MyD88)	NLRC4 (IPAF)	Bacterial flagellin and other components of the bacterial secretion apparatus	Cytoplasm	Trigger the secretion of IL-1β from neutrophils.	NF-κB Caspase-1	IL-1, IL-18	(98, 99)
	NOD1	Peptidoglycan (Gram-negative bacteria)	Cytoplasm	Recognition of intracellular bacterial cell products, Phagocytosis, Bacterial killing, neutrophil migration			(100, 101)
	NOD2	Peptidoglycan (Gram-positive bacteria)	Cytoplasm	Recognition of intracellular bacterial cell products, Phagocytosis, neutrophil migration, Bacterial killing, Degranulation			(100, 102)
	NOD5/NLRX1	dsRNA (viruses)		In response to TLR2 ligands, NLRX1 induces the production of neutrophil ROS.			(103)
	NLRP1	Muramyl dipeptide moiety of PGN (bacterial cell wall); Anthrax lethal toxin (Bacillus anthracis)		No neutrophil function described			(98, 104)
	NLRP3	PAMPs, virulence factor, DAMPs	Endosome	Response to multiple stimuli *via* forming a NALP3 inflammasome and secreting IL-1β, caspase-1 activation			(98, 100)
	NLRP6	Unknown		It negatively regulates TLR-induced canonical NF-kB and MAPK pathways in murine			(105, 106)
	NLRP12			It mediates the secretion of IL-1β induced by inflammasomes in neutrophils.			(107)

In order to ascertain the role of NOD-receptors in COPD, Barton et al. studied the expression of NOD1 in some alveolar epithelial type II cells, airway epithelial cells, endothelial cells as well as alveolar macrophages (109). They found that patients with chronic bronchitis had decreased expression of NOD1 in their lung tissues (109). DAMP-activated inflammasome may also have a contributing role in the pathophysiology of COPD. Conditions such as infections, hypercapnia, inhaled toxic agents, focal hypoperfusion, oxidative stress, tissue acidification, necrotic cell death and hypoxia may induce damaged lung tissues to release DAMPs (e.g., uric acid, ATP), leading to the activation of NLRP3 inflammasome. NLRP3 activation protects the host from infections caused by several pneumonia-causing bacteria such as *C. pneumoniae, S. pneumoniae, K. pneumoniae, S. aureus*, and *L. pneumophila* (110–112). Aged mice had decreased NLRP3 expression and function, which made them more susceptible to pneumonia, ALI, and death (113). Due to a diminished expression and function of NLRP3 in the lungs of an aging population, their susceptibility to secondary pneumonia caused by *S. pneumoniae* was enhanced (114). NLRP3 also increases the incidence and mortality rate of ALI (115). Decreased expression of NLRP3 also inhibited the onset of severe necrotic pneumonia caused by *S. aureus* by enhancing bacteria clearance (116). When NLRP3 is activated by α-hemolysin during *S. aureus-*induced pneumonia, it causes necrotizing pneumonia or necrotic lung injury that is independent of IL-1β signaling (116, 117). *S. aureus*-induced pneumonia does not only stimulate NLRP3 inflammasome but also stimulates NLRC4 inflammasome which induces necroptosis by inhibiting IL-17A-induced neutrophil trafficking to the lungs and the production of IL-18 (118). NLRC4 deficiency promotes neutrophil infiltration in the lungs, reduces necroptosis, improves pathogen clearance, and improves host survival. Thus, NLRC4 deficiency in both hematopoietic and non-hematopoietic cells protects the host from *S. aureus*-induced pneumonia (118). Activation of NLRC4 stimulates the production of IL-1 β, IL-17A, and neutrophil chemoattractants in the lung, which prove beneficial to the host during pneumonia caused by Gram-negative bacteria such as *K. pneumoniae* and *P. aeruginosa* (119). NLRC4 activation, on the other hand, causes inflammatory lung injury, increases lung bacterial burden, and causes necroptosis during *P. aeruginosa*-induced pneumonia (120). Furthermore, following *S. aureus* infections, NLRC4 suppressed IL-17A-dependent neutrophil accumulation by triggering necroptosis and IL-18 activation in the lungs (118). NLRP6-/- mice were more resistant to pulmonary infection caused by *S. aureus* than their wild-type counterparts, as they recorded improved survival rates and increased bacterial clearance in the lungs (121).

TLR2, TLR4, and NLRP3 expressed by mRNA in patients with COPD were significantly increased in neutrophils during acute exacerbation compared to stable disease. However, TLR9 expression by mRNA did not differ significantly between stable disease and exacerbation. Increased expression of TLR2, TLR4, and NLRP3 on neutrophils could make peripheral blood neutrophils more sensitive to DAMPs generated during COPD exacerbations. This means that, elevated TLR2 and TLR4 expression in neutrophils, along with higher DAMP levels, may contribute to DAMP-induced neutrophilic airway inflammation during COPD exacerbation (122). TLR2 and TLR4 are not receptors for only DAMPs but also pathogen-associated molecular patterns (PAMPs). Thus, PAMPs may play a role in the inflammatory response during airway infection-associated exacerbations. ATP, a recently classified DAMP, is known to activate NLRP3 and has been shown to be elevated in BAL fluid of COPD patients (123). Pro-inflammatory cytokines IL-1 and IL-18 are released when NLRP3 is activated on neutrophils, and this has been linked to the development of COPD (123, 124). Exposure to cigarette smoke extract is known to increase TLR4 expression in bronchial and nasal epithelial cells during pulmonary inflammation (125, 126).

Airway epithelial cells recognize different pathogens during pneumonia due to the expression of various PRRs. These PRRs can be either extracellular TLRs (TLR1, TLR2, TLR4, TLR5, and TLR6), intracellular TLRs (TLR3, TLR7, TLR8, and TLR9) or NLRs inflammasome (127–129). In response to TLR activation during *K. pneumoniae*-induced pneumonia, TRIF signaling pathway can provide some antibacterial defense by inducing interferon (IFN)-x03B3 in the lungs (85). Due to the attenuation of neutrophil sequestration and the production of MIP-2, TNF-, IL-6, and LIX, Toll/IL-1R Domain-Containing Adaptor Protein (TIRAP), has been reported to play a critical role in pneumonia caused by *K. pneumoniae* (130). During a *P. aeruginosa*-induced pneumonia, it was evident that TIRAP is not required for neutrophil infiltration, LIX production, and bacterial clearance (130). Also, TLR2-induced MyD88 activation was not required for the clearance of *S. aureus* during pneumonia. However, TLR2-induced MyD88 activation is known to trigger an important inflammatory immune response and it is very critical for the clearance of *P. aeruginosa*-induced pneumonia (131). When compared to airway neutrophils from healthy subjects, a large fraction of neutrophils isolated from the BALF of patients suffering from chronic airway inflammation had upregulated levels of TLR2, TLR4, TLR5, and TLR9, as seen in CF and non-CF-bronchiectasis. These changes are associated with neutrophil respiratory burst activity, and is concomitant with *de novo* protein synthesis, granule exocytosis, and later induction of apoptosis (21, 96, 132). For mucosal intrinsic defense activity against non-pathogenic *E. coli, S. enterica, S. pneumoniae*, and *P. aeruginosa*, TLR5 has been confirmed to play such critical role in a rodent model (133, 134). TLR9-deficient mice are unable to produce Th1 effector cells, resulting in a higher bacterial load in the lungs (135). Thus, TLR9 plays a detrimental role in pneumonia caused by both *P. aeruginosa pneumonia and methicillin-resistant S. aureus* (136).

## Roles of Chemokines in Lung Neutrophil Immunity

Chemokines have a low molecular weight that ranges from 7 to 15kDa and are the largest family among small cytokines. Together with their receptors, chemokines are able to regulate the migration and residence of all immune cells. Although some chemokines are considered pro-inflammatory because they are influenced by immune reactions, others are considered homeostatic and operate to regulate the migration of cells during tissue growth and repair. Chemokines are very important because of their specific physiological role, i.e. they induce the recruitment of specific subset of leukocytes (137, 138). Through subtraction hybridization process, several chemokines were originally recognized as early response genes that are stimulated by growth factors. The assumption was that, chemokines, based on this property, acted as nuclear factors and took part in the proliferation of cells. However, based on complete amino acid sequencing, chemokines were confirmed as secretory proteins. CXC chemokines: CXCL1-8 and CXCL12, and CC chemokines: CCL2, CCL17 (TARC), CCL18 (PARC), and CCL20, are the most important chemokines involve in the recruitment of neutrophils into the airways (**Table 2**) (151). Different cytokines produced by local airway cells (Thymic stromal lymphopoietin and IL-33, IL-25, IL-23, IL-17, IL-10, IL-1-β, IL-1-alpha), are those that transmit the relevant biological impact of chemokines.

**Table 2:** Main murine and human chemo-attractants and their receptors expressed in neutrophils during pulmonary infection and inflammation.

Chemo-attractants			Receptors		Ref
Systemic name	Name in Human	Name in Murine	Human neutrophils	Murine neutrophils	
CXCL1	GROα	KC	CXCR2	CXCR2	(139, 140)
CXCL2	GROβ	MIP-2	CXCR2	CXCR2	(141)
CXCL5	ENA-78	LIX	CXCR2	CXCR2	(142, 143)
CXCL6	GCP-2	NA	CXCR1/CXCR2	NA	(144)
CXCL8	IL-8	NA	CXCR1/CXCR2	CXCR2	(65, 145)
CXCL12	SDF-1α	SDF-1α	CXCR4	CXCR4	(146)
CCL3	MlP-lα	MlP-lα	NA	CCR1	(147, 148)
CCL5	RANTES	RANTES	NA	CCR1	(140, 149)
CCL7	MCP-3	MARC	NA	CCR1	(150)

NA, not applicable.

**Figure 4:**
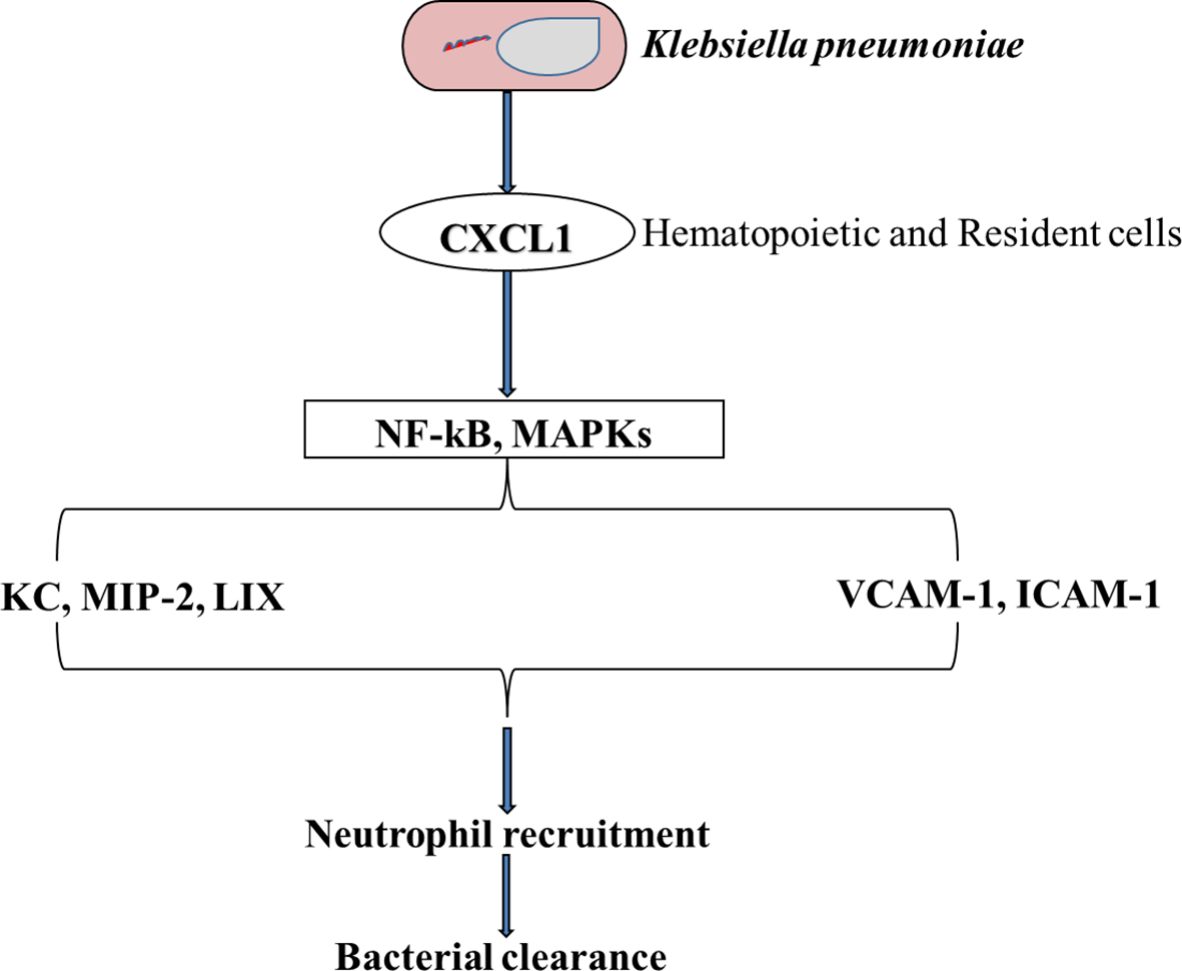
A Schematic representation of the importance of CXCL1 in neutrophil influx when the lung is challenged with *K. pneumoniae*. CXCL1 influences the activation of both NF-kB and MAPK which leads to an increase in MIP-2 and LIX chemokines, and other adhesion molecules such as VCAM-1 and ICAM-1. This cascade of events leads to an influx of the lungs with neutrophils followed by the clearance of the bacteria (154).

Both CXC and CC chemokines play important roles in the stimulation of neutrophil chemotaxis. Under normal conditions, CXCL1 levels are negligible, however, they increase substantially during active infections in the lungs. According to Paudel et al., Cxcl1^-/-^ mice showed impaired neutrophil recruitment and poor bacteria elimination from their BALF and lungs when challenged with a *S. pneumoniae* (152). In a pneumococcal infection of the lungs, CXCL1 was known to regulate neutrophil recruitment through a CD62L- and CD49d-dependent process. Results from Batra et al. showed that CXCL1 is an important chemokine for neutrophil influx and the production of Leukotriene B4 (LTB4) in the lungs during *K. pneumoniae* infection and is also very important for ROS regeneration in the lungs (153). Their analysis also validated the significance of CXCL1 in NADPH oxidase expression and the formation of NO and free radicals of oxygen in neutrophils after *K. pneumoniae* infections in the lungs. Studies have shown that CXCL1 is essential in NF-kB activation within the lungs after *K. pneumoniae* infection (**Figure 4**). NF-kB protects the lungs by avoiding excessive injury and inflammation during pneumococcal and *E. Coli*-induced pneumonia (155, 156). NF-kB has again been shown to be important in antibacterial host defense (157, 158) and hence reiterating the importance of CXCL1 in neutrophil recruitment (154). After infection with an influenza A virus (IAV), CXCL1, CXCL2, and neutrophils were found in lung tissues and airways of neonatal mice (159, 160). Furthermore, non-endothelial cells, non-epithelial cells (ATII cells) and lung stromal cells were shown to be important for the induction of Cxcl1 in a MyD88/TRIF signaling dependent manner during infection with a respiratory syncytial virus (RSV) (161). The precise source of neutrophil chemoattractants during viral infection is an important research topic for future targeted neutrophilic inflammation therapies. During viral infections, many neutrophil chemoattractants such as CXCL1, CXCL2, and IL-17 (161, 162) are produced in the lungs and airways, which cause neutrophil trafficking into the lungs of mice and ferrets (161, 163). By the activation of CXCR2 on neutrophils and their interaction with GAGs, CXCL2/3 chemokines can orchestrate the recruitment of neutrophils to the lungs during pulmonary infections. By binding to its receptor (CXCR2), CXCL5 chemokine, also known as lipopolysaccharide-induced chemokine (LIX), plays a significant role in the trafficking of neutrophils to the lungs during infection and inflammation (57). According to Gibbs et al., clock-controlled-CXCL5 mediates circadian variation and activates the rhythmic recruitment of neutrophils to the lung during pulmonary infection (164). CXCL8, also known as IL-8, is one of the most effective chemo-attractants (165), which can bind to the G protein–coupled receptors CXCR1 and CXCR2 on neutrophils (166). One of its numerous functions is to guide neutrophils through the tissue matrix until they reach the inflammation site. By using different signaling mechanisms, CXCL8 induces specific intracellular signaling cascades that result in rapid neutrophil recruitment (167–170). CXCL8 influences the movement of neutrophils across the endothelium (171), pulmonary epithelium (172), and fibroblasts (173). CXCL-12 is expressed in the lungs by cells such as the endothelial and epithelial cells (146). Both the ligand (CXCL12) and its receptor (CXCR4), play an important role in neutrophil influx to the lungs during infection (174–176). The signaling pathway of CXCL12/CXCR4 plays a crucial role in modulating neutrophil action in ALI not only by enhancing chemotaxis but by preventing cell death. CXCR4 inhibition reduces the trans-endothelial and trans-epithelial trafficking of neutrophils in pulmonary inflammation (175). Following a tissue damage, there was an increase in the local production of CXCL12; an important chemokine in the reparative cascade, which resulted in the guidance and recruitment of stem cells to the lungs (173). Although some studies have iterated the role of CXCR4 in the augmentation of pulmonary fibrosis (177), others have also reported on their role in neo-alveolarization (178). In a recent study, it was also discovered that CXCR4^hi^ neutrophils are more likely to induce NETs, which increase the uptake of house dust mite by inflammatory dendritic cells, thereby increasing the risk of allergic asthma (179). The overexpression of neutrophil chemoattractants such as CXCL1, CXCL2, CXCL3, CXCL5, IL-8 (CXCL8), and CCL20 in the lungs of COVID-19 patients suggest that these cells can express neutrophil chemokines after SARS-CoV-2 infection (180). Chemokines also play important roles in metastasis and apoptosis. It has been shown that CXCL1 promotes cell migration. Because of this, Guo et al., performed a cell-proliferation inhibitory experiment by using Jinrong granule. Their results showed that Jinrong granule could inhibit the ability of CXCL-1 to promote the migration and proliferation of breast cancer cells and it could also reverse the promoting effect of CXCL-1 on breast cancer through the CXCL-1- CLCR2/CCL20 pathway (181). By using this analogy, therapies can be designed to target the cell migratory and proliferation potential of CXCL-1 during pulmonary infections and inflammation.

Some CC chemokines are classified as inflammatory (CCL2, CCL3, CCL4, CCL5, CCL11 and CCL13) while others are classified as homeostatic (CCL18, CCL19, CCL21, CCL25 and CCL27). However, some are considered as having both homeostatic and inflammatory function (CCL14, CCL15, CCL16 and CCL23) (182). Under normal conditions, there is no expression of CC chemokine receptors (CRs) (183), and these receptors do not respond to their CC chemokine ligand even upon stimulations. However, it has been shown that under inflammatory conditions in the lungs, neutrophils after their migration, expand their CR expression repertoire (21, 184). Functions of neutrophils which include chemotaxis, phagocytosis and respiratory bust are usually altered when there is an induction of CC chemokines and their receptors (CRs) (21, 184). The expression of these receptors are modulated by pro-inflammatory cytokines including IFN-γ, TNF-α, and GM-CSF. Macrophage inflammatory protein 1 (MIP-1/CCL3), which is one of the important members of the CC chemokine family, mediates the development of neutrophils and regulates their recruitment to the lungs during infection. MIP-1 has been reported to be chemotactic for neutrophils (185). In a study by Bonville et al., it was confirmed that neutrophil recruitment to the lung parenchyma in response to heterologous CCL3 expression in the respiratory epithelium, is directly dependent on IFNγ signaling (186). During a *S. pneumoniae*-induced inflammation, CCL2 chemokine binds to its receptor (CCR2), which triggers a PI3Kγ-dependent downstream signaling cascade, resulting in neutrophil immigration into the lung (187, 188). Also, CCL2 and CCL7 chemokines are known to work synergistically with CXCL8 to drive neutrophil trafficking to the lungs during acute respiratory distress syndrome (ARDS) (150). Grommes et al., indicated that the recruitment of neutrophils during an LPS-, acid-, and sepsis-induced ALI, is made possible because of the release of CCL5-CXCL4 heterodimer from platelets, and any disruption of this heterodimer decreases the amount of neutrophils recruited to the lungs (189). CCR1, CCR2, CCR3, CCR5, CXCR3, and CXCR4 were upregulated by a large fraction of neutrophils isolated from the BALF of patients suffering from chronic airway inflammation, as seen in CF, COPD, and asthma (190).

## Neutrophil Oxidative Burst

Oxidative burst is a critical antimicrobial mechanism of neutrophils and is mediated by nicotinamide adenine dinucleotide phosphate (NADP) oxidase (191). Despite having the ability to protect against pulmonary infection, neutrophils, if left uncontrolled, can cause pathogenic effects through a variety of functions (192). Reduced NADP (NADPH2), thiocyanate, ergothioneine, thiosulfate, reduced glutathione (GSH), reduced nicotinamide adenine dinucleotide (NADH2), azide, Tapazole, thiourea, cyanide, cysteine, and tyrosine are the main components of the myeloperoxidase system, which performs this antibacterial activity. Genetic mutations in the NADPH oxidase subunit, gp91 (also referred to as NOX2), are associated with chronic recurrent and life-threatening microbial infections. When neutrophils are stimulated by microbes or by integrin-dependent adhesion to the ECM, they release reactive oxygen intermediates (ROIs). In mice, both the Vav family of Rho GTPase guanine nucleotide exchange factors (GEFs) and phospholipase C–γ2 (PLC-γ2) have been shown to be critical mediators of adhesion-dependent ROI production by neutrophils. Vav is critical for neutrophil-dependent host defense against *S. aureus- and P. aeruginosa-*induced hospital-acquired pneumonia (193). Compared to healthy controls, PMBCs and erythrocytes isolated from patients with tuberculosis had a significantly decreased levels of GSH (194). However, elevated levels of GSH have been shown to improve the inhibition capacity of T-cells against the growth of *M. tuberculosis* during pulmonary inflammation (195).

During oxidative burst, neutrophils produce reactive oxygen specie (ROS) which has been shown to induce necrosis cause by *M. tuberculosis* (196). Rapid assessment of neutrophil oxidative burst capacity has been proposed as an effective way to identify patients at risk of excessive immune responses during pulmonary inflammation (197). Therefore, the correlation between GSH and/or NADPH2 levels in TB patients and neutrophils oxidative burst capacity can provide host-targeted therapies. While some authors have reported an increased ROS production by blood PMN in cystic fibrosis (198, 199), others reported that the production of ROS is dependent on the pathogenic agent (200) or the detection method employed to measure the respiratory burst activity (201). Montemurro et al. (202) have demonstrated that blood neutrophils of CF patients had higher ROS release than their control counterparts. Lung ischemia-reperfusion injury has been linked to the production ROS and oxidative burst (203).

## Pathophysiological Role of Neutrophils, NETs in COVID-19

Numerous studies have suggested that the recruitment of neutrophils to the lungs is linked to disease severity during viral infection. During an RSV-induced severe bronchiolitis, neutrophils accounted for nearly >90% of the BAL cell composition, confirming the role of neutrophils in disease pathogenesis (204, 205). Elevated levels of neutrophils and their markers in the lungs have also been observed in both rhinovirus and hMPV-infected children and in severe cases of influenza and SARS-CoV-2 infection (206, 207). Elevation in neutrophil level is one common phenomena observe during severe respiratory viral infections, and it is reasonable to postulate that their recruitment to the lungs and subsequent activation can exacerbate tissue pathology and cause disease. The current global pandemic, COVID-19, is a multisystem inflammatory disease caused by the SARS-CoV-2 virus. The innate immune response has been widely linked to COVID-19 immunopathogenesis. After reaching the alveoli, SARS-CoV-2 activates alveolar macrophages, which induces innate immune responses. A complement cascade is then activated by the viral particles through the lectin pathway. C3a and C5a are complementary peptides which are generated after the activation of the complement system. This then stimulate the migration of neutrophils to the site of infection. SARS-CoV-2 S-protein induces the release of proteins such as epithelial membrane protein 2 (Emp2) by the lung epithelial cells. The Emp2 of alveolar epithelial type 1 cells upregulate neutrophil migration. COVID-19 pathogenesis has been linked to neutrophil infiltration into the lungs. However, the numerous functions of neutrophils which include its interaction with other immune cell population, virus internalization and killing, cytokines release, degranulation, oxidative burst, and the production of neutrophil extracellular traps (NETs), helps to improve antiviral defenses (208, 209). Degranulation and the activation of neutrophils are the highly activated processes in SARS infection (210).

Neutrophilia has been identified as one of the markers linked with poor prognosis and severe respiratory symptoms in COVID-19 patients (211–213). According to Wang et al., neutrophilia is associated with lung injury in patients with severe COVID-19 (214). During autopsies of COVID-19 victims, neutrophilic mucositis was found in the lungs, indicating that the entire LRT was inflamed (215, 216). By using a Myeloperoxidase (MPO), Neutrophil Elastase (NE) and a Citrullinated Histone H3 (citH3) staining method, neutrophil infiltration *via* neutrophilic plugs was detected in patients with COVID-19 (217). Similarly, elevated levels of neutrophils have been found in peripheral blood of both severe and non-surviving COVID-19 patients (218, 219). According to a research by Parackova et al., neutrophils enhance the stimulation of Th17 in patients with COVID-19, and these cell population have been implicated in immune-mediated injury (220). It has been revealed that the infiltration of lungs with immature and/or dysfunctional neutrophils, as defined by the expression of CD11b, CD16, CD24, CD34, and CD38 and the infiltration with recently activated neutrophils which is characterized by the expression of CD64, RANK, RANKL and reduced CD62L, have been implicated as the causes of imbalance immune response during severe COVID-19 cases (220, 221). Severe COVID-19 pathophysiology is also characterized by altered neutrophil quantity, phenotype, and neutrophil functioning. Increased numbers of neutrophils have been reported in the nasopharyngeal epithelium (222) and later in the more distant regions of the lung following SARS-CoV-2 infection (223). An increase in the number of neutrophils has also been detected as a characteristic feature in the blood of COVID-19 patients (224–226) and markers of neutrophil activation are an important feature of blood transcriptomes in severe cases (227, 228). Increased levels of CXCL2 and CXCL8 have been shown through Transcriptional analysis of peripheral blood mononuclear cells and BALF from COVID-19 patients, to contribute to the recruitment of neutrophils to the lung, which then exacerbate the inflammatory response (206). Moreover, activated neutrophils express properdin, factor B, and C3, thus driving complement activation (229), a marker of severe COVID-19 disease (230, 231).

Infiltration is not the only mechanism by which neutrophils cause pathology in COVID-19 patients. Indeed, several inflammatory conditions including thrombosis, sepsis, and respiratory failure have all been linked to the pathological effects of NETs (232–234). A disproportionate release of virus-induced NET have been reported in COVID-19 patients and this is linked to the pathogenesis of this disease. The release of NETs by neutrophils has been linked to organ damage and death in COVID-19 patients (215). Another recent study found that markers of NET release (myeloperoxidase-DNA and citrullinated histone H3) in the sera of COVID-19 patients potently triggered NETosis in control neutrophils (235). COVID-19 patients have elevated levels of IL-6, which is a likely driver of NETosis. Similar in other inflammatory diseases, IL-6 stimulates the release of NETs throughout the body of COVID-19 patients (236, 237). Virus-damaged epithelial cells (56, 238), activated endothelial cells (239), activated platelets (240, 241), and inflammatory cytokines like IL-1β are all triggers of NETosis (242, 243). Higher levels of NETs have been seen in COVID-19 patients (235, 244, 245), and an increase in plasma NETs has been linked to increased COVID-19 severity (245), lung damage and microvascular thrombosis (244). Uncontrolled NETs can potentially trigger thrombosis.

## Pathological Role of Neutrophils in Thromboembolism

**Figure 5:**
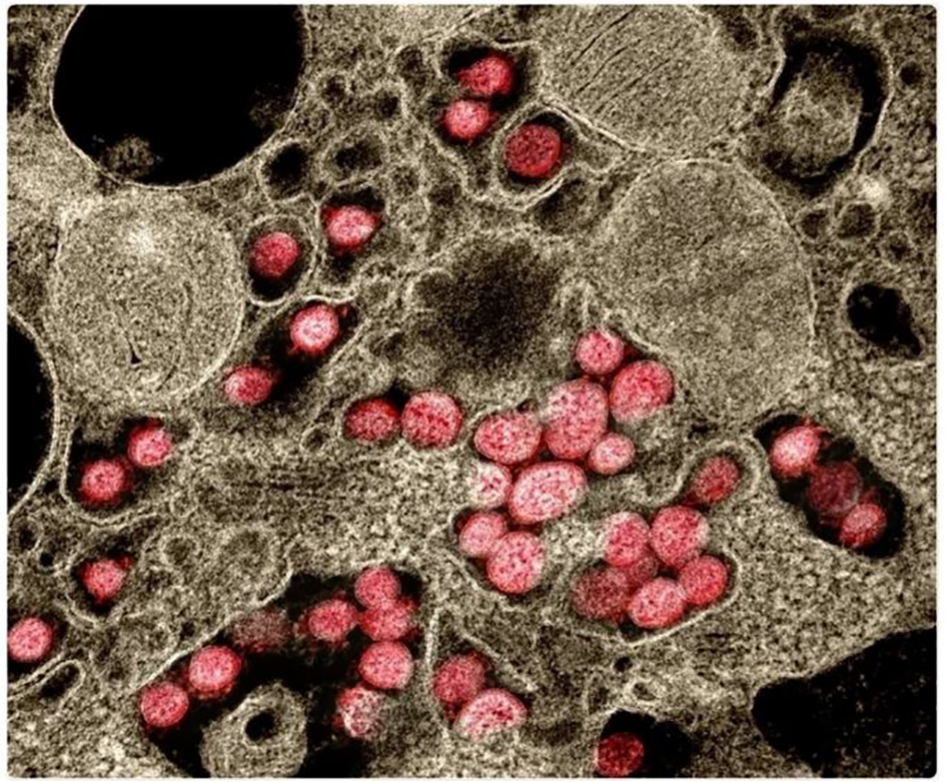
Neutrophil rather than platelet activation are associated with thrombotic complications in COVID-19 patients (246).

Rather than platelets, neutrophils can play the lead role in thrombotic complications associated with COVID-19 (**Figure 5**). NETs have been demonstrated to exert thrombogenic activity in several inflammatory diseases by expressing functionally active tissue factor (TF). Thrombin-antithrombin (TAT) activity which indicates the activation of a TF/thrombin axis has been strongly linked with the levels of myeloperoxidase (MPO)/DNA complexes. NET release has also been identified as a key contributor to neutrophil-related thromboinflammation, providing the scaffold for platelet entrapment and activation. An *in vitro* and ex vivo models have been used to demonstrate the role of NETs in neutrophil-related thromboinflammation (247, 248). Leppkes et al. concluded that the development of NETs inside the microvessels of patients suffering from COVID-19 is associated with the severity of the disease. Rapid vessel occlusion caused by the intravascular development of NETs with platelet aggregation results in organ damage (249). In a study by Nicolai et al., it was noted that the kidney, lung, and heart of patients with COVID-19 had inflammatory microvascular thrombi which contained NETs and platelets (250). Thrombotic complications contribute to morbidity and mortality in severe COVID-19 (251, 252). In COVID-19 patients, abnormal coagulation parameters and elevated levels of proinflammatory cytokines are correlated with disease severity, poor prognosis, and incidence of venous thromboembolism. Thrombosis affects circulation in both the veins and the arteries of patients with COVID-19, leading to deep vein thrombosis, acute coronary syndrome, pulmonary embolism, stroke and microvascular thrombosis (253, 254). NET-remnants, such as citrullinated H3, circulating cell-free DNA, or MPO-DNA complexes have been found in abundance in the blood of COVID-19 patients (244, 249). Furthermore, patients with severe illness had elevated levels of both neutrophil activation markers and neutrophil-platelet aggregates (250, 255). Importantly, tissue factor is abundant in NETs from COVID-19 patients (TF). The release of thrombogenic NETs decorated with TF have been associated with activation of the complement system (256). Vascular injury is one of the outcome of excessive formation of NETs (257). Excessive NET can leads to the formation of autoantibodies that determine the appearance of various forms of autoimmune vasculitis (258). Immunothrombosis associated with NETs release has been shown *via* a histopathological study, to be linked to organ damage in severe COVID-19 (259). Occlusion of small pulmonary vessels caused by aggregated NETs was found in lungs during autopsies from victims of COVID-19-related ARDS (244). In a K18-hACE2 transgenic mice infected with SARS-CoV-2, neutrophils were seen infiltrating the alveolar and interstitial areas, which resulted in severe pulmonary pathology (162). Aggregated NETs may clog microvessels and this contribute to poor outcomes in COVID-19. DNAses prevent vascular occlusions which are caused by non-canonical NET-driven thrombosis during a steady-state condition (260). Thisfinding suggests that NET-dissolving mediators in patients can be impaired or elevated (261). In a quest to understand the role of neutrophil-lymphocyte in SARS-CoV-2 infection, Nicholai and his team compared histopathological lung specimens of COVID-19 with that of a viral pneumonia caused by H1N1 or seasonal influenza virus. Their findings highlighted neutrophil-driven immunothrombosis as a key element of severe COVID-19, as immunothrombotic vessel occlusion and NETosis were strongly elevated compared to influenza pneumonia (262).

## Neutrophilia and Neutrophils in Lung Destruction and Resolution

Increased pulmonary vasculature permeability, accumulation of neutrophils in alveoli and disruption of the alveolar epithelium are all characteristic features of acute inflammatory response (263). Alteration in alveolar function i.e. leakage of plasma and interstitial fluid into airspace, is linked to damaged alveolar epithelial cells caused by transmigration of neutrophils from the alveolar capillaries to the airspace. Excessive neutrophils can cause tissue damage by increasing inflammatory response and by directly releasing toxic effectors. At high concentrations, many neutrophil effector mediators can cause tissue damage. For instance, although NE plays a role in digesting extracellular matrix (264) and induces mucus production (helps in pathogen clearance), when produced in excess, it can contribute to airway pathology because mucus plugs can obstruct the airways (265). The combination of mucus production and NET release can damage tissues and impair lung function (56, 266). Increase in blood neutrophil levels can predict severe respiratory damage and is an early stage marker for SARS-CoV-2 infection (267, 268). Thus, cytokines release and respiratory failure may be as a result of neutrophilia and excessive NETs. Excessive NETs can directly kill epithelial and endothelial cells (191, 269), damage the epithelium in pulmonary fungal infection (270) and cause severe injury to the endothelium during acute lung injury (271).

However, some researchers have opined that transmigration of neutrophils can occur without any destruction to major barriers and that, neutrophil accumulation can lead to the repair and regeneration of lung epithelium. The ability of accumulated neutrophils to repair tissues is partly due to the clearance of epithelial debris from the damaged sites which creates a clean matrix for regeneration of the epithelium (272). Furthermore, neutrophils can induce a repair response by activating the proliferation of lung epithelial cell (273) and by secreting pro-resolution products such as Annexin A1 (274). Neutrophil transmigration through an elastase-mediated cleavage of E-cadherin has been shown to activate the β-catenin signaling in alveolar type II epithelial cells in mice treated with keratinocyte chemokine or intratracheal LPS (275). In an acid-induced acute lung injury model, neutrophilia was essential for the proliferation of type II pneumocytes, which are necessary in regenerating alveolar epithelium. Neutrophilia, according to proteomic analysis, promote multiple regenerative pathways, including MMP9, MMP2, and FGF1 (276).

## Diagnostic Neutrophilic Biomarkers of Pulmonary Inflammations

Biomarkers have been defined as “a characteristic that is objectively measured and evaluated as an indicator of normal biological processes, pathogenic processes, or pharmacologic responses to a therapeutic intervention”. Many neutrophilic biomarkers associated with pulmonary inflammation have been studied (**Table 3**). The large number of biomarkers studied reflect the complex pathophysiology of pulmonary inflammations and the heterogeneity of the host response. This portion of the review summarizes some of these molecular biomarkers.

**Table 3:** Neutrophilic biomarkers of pulmonary inflammations.

Type of Pulmonary inflammation	Biomarkers	Tissue/Detection level	Ref	Type of Pulmonary inflammation	Biomarkers	Tissue/Detection level	Ref
**ARDS**	Von-Willebrand Factor	Plasma/ 351% ± 265%	(277)	**Pneumonia**	CRP (mg/L)	fingerstick blood/ 60 (18-134)	(278)
	SP-D (ng/ml)	Plasma/ 275 (80 – 462)	(279)		PCT (ng/ml)	Serum/ 3.64 ± 12.32	(280)
	LDH (IU/L)	Serum/ 274 ± 104	(281)		sTREM-1 (pg/ml)	Serum/ 183.9 (119.8-232.1)	(282)
	TNF-a (pg/mL)	Plasma/ 7.5 (3.8–13.4)	(283)		ProADM (nmol/L)	Venous blood/ 2.341 (1.188-4.226)	(284)
	IL-6 (pg/mL)	Plasma/ 240 (139-498)	(283)		IL-6 (pg/mL)	Serum/ 242.2(92.33-473.97)	(285)
	IL-10 (pg/mL)	Plasma/ 77 (31-169)	(283)		Angiopoietin-2 (ng/ml)	Serum/ 5.92 (3.48–9.99)	(286)
	Protein C	Pulmonary edema fluid and plasma/ 37% ± 14%	(287)		Presepsin (pg/mL)	Plasma/ 1734 (1014-3128)	(288)
	Plasminogen Activator Inhibitor (ng/ml)	alveolar fluid and plasma/ 2687 ± 1498	(289)		Calprotectin (mg/L)	Serum/ 7.43 (4.60, 10.33)	(290)
	CC16 (ng/ml)	Plasma/ 14.3 (9.0 - 19.0)	(279)		FGF21 (pg/mL)	Serum/ 456.5(181.2−1127.9)	(291)
	KL-6 (U/l)	Plasma/ 477 (287-636)	(279)		NETs (U/mL)	BALF/ 223 (40.6–766)	(292)
	sRAGE (ng/ml)	Plasma/ 1932 (960-4267)	(293)		PTX3 (ng/ml)	BALF/ ≥1	(294)
	sE-selectin (ng/mL)	Serum/ 53.0 ± 17.8	(281)		TNF-α (pg/mL)	Plasma/ 44 (37–62)	(295)
	CRP (mg/dL)	Serum/ 18.6 ± 11.0	(281)		D-dimer (mcg/L)	Plasma/ 2080 (1050–3410)	(296)
**COVID-19**	(MPO)-DNA (AU)	Serum/ 1.31 ± 0.18	(297)				
	NE (ng/mL)	Plasma/ 144 (84–248)	(298)				
	NLR	Serum/ 8.78 (5.76-25.10)	(299)				
	TAT (µg/mL)	Plasma/ 7.30 (4.50-12.2)	(300)				
	VWF	Plasma/ 306% (200-421)	(300)				
	ADAMTS13	Plasma/ 47.3% (25.8-66.1)	(300)				
	PAP (ng/mL)	Plasma/ 984 (648-2377)	(300)				
	CRP (μg/L)	Serum/ 81.49 (19.40-107.42)	(299)				

ADAMTS13: a disintegrin and metalloproteinase with a thrombospondin type 1 motif, member 13; CRP, C-reactive protein; CC16, Clara cell secretory protein; FGF21, fibroblast growth factor 21; IL-6, interleukin-6; KL-6, Krebs von den Lungen-6; LDH, lactate dehydrogenase; MR-proADM, midregional-proadrenomedullin; (MPO)-DNA, myeloperoxidase (MPO)-DNA; NE, neutrophil elastase; NLR, neutrophil-to-lymphocyte ratio; NETs, neutrophil extracellular traps; PAP, plasmin-antiplasmin complex; PCT, procalcitonin; PTX3, pentraxin 3; sRAGE, soluble receptor of advanced glycation end products; SP-D, surfactant protein; sE-selectin, soluble endothelia-selectin; sTNF, soluble tumor necrosis factor receptors TNF-α: tumour necrosis factor alpha; TAT, thrombin-antithrombin complex.

Alpha-1 antitrypsin and CD16b (AAT : CD16b) protein complex released by primed neutrophils has been found to be significantly elevated in sera of patients with CF, making it a potential biomarker to diagnose exacerbated cystic fibrosis. The expression of these neutrophil priming-associated biomarkers in peripheral blood can be used to expound the inflammatory process in CF (301). Adenosine monophosphate and purines adenosine triphosphate have also been identified as potential neutrophilic biomarkers of pulmonary inflammation in CF. In chronic obstructive pulmonary disease, research have shown that, the development of systemic inflammation (302–305), exacerbations and other lung functioning parameters (306, 307) are positively linked with the levels of fibrinogen, C-reactive protein, and IL-6. COPD exacerbation has been linked to elevated levels of neutrophil gelatinase associated lipocalin (NGAL), osteoprorotegerin and soluble TNF receptor-1 (sTNFR-1) (308, 309). Additionally, vascular endothelial growth factor (VEGF) and MPO were predicting factors in determining the severity of lung function impairment and dyspnea (310). There have been numerous studies reporting decreased concentrations of Clara cell protein (CC-16) in the blood of people suffering from COPD, supporting the notion that this protein may be an important biomarker in the prediction of bronchial epithelial cell dysfunction (311–315). Compared to their control counterparts, people with COPD had elevated levels of NGAL, heparin-binding EGF-like growth factor (HB-EGF), extracellular newly identified RAGE-binding protein (EN-RAGE; also known as S100A12), MPO, fibrinogen and transforming growth factor alpha (TGF-α). Conversely, COPD patients had lower levels of soluble receptor for advanced glycation end products (sRAGE) than the control group (316). Active neutrophil elastase (NE), a serine proteinase secreted by neutrophils in response to inflammation and pathogen invasion is elevated during exacerbations of COPD and may be a viable biomarker for distinguishing a bacterial exacerbation in patients with COPD (317). A number of biomarkers have been used as predictive biomarkers for pneumonia. These include copeptin, CRP, proadrenomedullin (proADM), and procalcitonin (PCT) (318–322). Calprotectin, PCT, plasma pentraxin 3 (PTX3) and presepsin have been used as promising acute-phase molecular predictive markers for community-acquired pneumonia (323). von Willebrand factor (VWF), adhesion molecules (such as E-selectin, L-selectin, intercellular adhesion molecule [ICAM], and vascular cell adhesion protein-1), thrombomodulin (TM), protein C, and plasminogen activator inhibitor-1 (PAI-1) are also known as important markers of endothelial activation and injury in acute respiratory distress syndrome (ARDS). A number of coagulation biomarkers including protein C, thrombomodulin and PAI-1 have been shown to be abnormal in ALI (324). The epithelial mucin protein, Kerbs von den Lungren-6 (KL-6), has also been studied as a potential biomarker. KL-6 is unregulated when type II pneumocytes become injured (325).

Inflammation and immunity play a critical role in many chronic diseases. Neutrophil-lymphocyte ratio (NLR) is a biomarker that reflects the balance between acute and chronic inflammation (neutrophil count) as well as adaptive immunity (lymphocyte count). It can be computed as the ratio between neutrophils and lymphocytes in peripheral blood. NLR, as determined by a recent meta-analysis (326), appeared to be a predictive biomarker for acute exacerbations in patients with COPD. In patients with COVID-19 infection, NLR was shown to have a good predictive value on disease severity and mortality (327). Data from recent studies suggest that NLR is an important predictor of mortality among patients with the novel Coronavirus disease (79). Numerous studies have implicated NLR in the development of COPD. In an examination of acute episode of COPD, Lee et al. reported that the NLR in the acute episode was significantly higher than that in the stable period and in the healthy control group. However, the NLR significantly decreased in the recovery stage patients with acute exacerbations (328). According to Taylan et al, NLR gradually increased with the severity of COPD, suggesting that NLR can be used as an early biomarker of COPD (329). The results of these studies confirm that NLR has great value in the assessment of COPD severity and acute exacerbations. Although not related to pulmonary inflammation, Huang et al., through a research to explore the applicative value of preoperative NLR combined with serum carcinoembryonic antigen (CEA), carbohydrate antigen (CA) 19-9, CA 125 and CA 72-4 levels, proved that NLR can reflect the inflammatory and immune status in gastric cancer patients and that, these combinations are closely related to clinical pTNM stage in gastric cancer (330).

## Clinical Trials Targeting Neutrophils in Neutrophil-Driven Pulmonary Inflammatory Diseases

**Figure 6:**
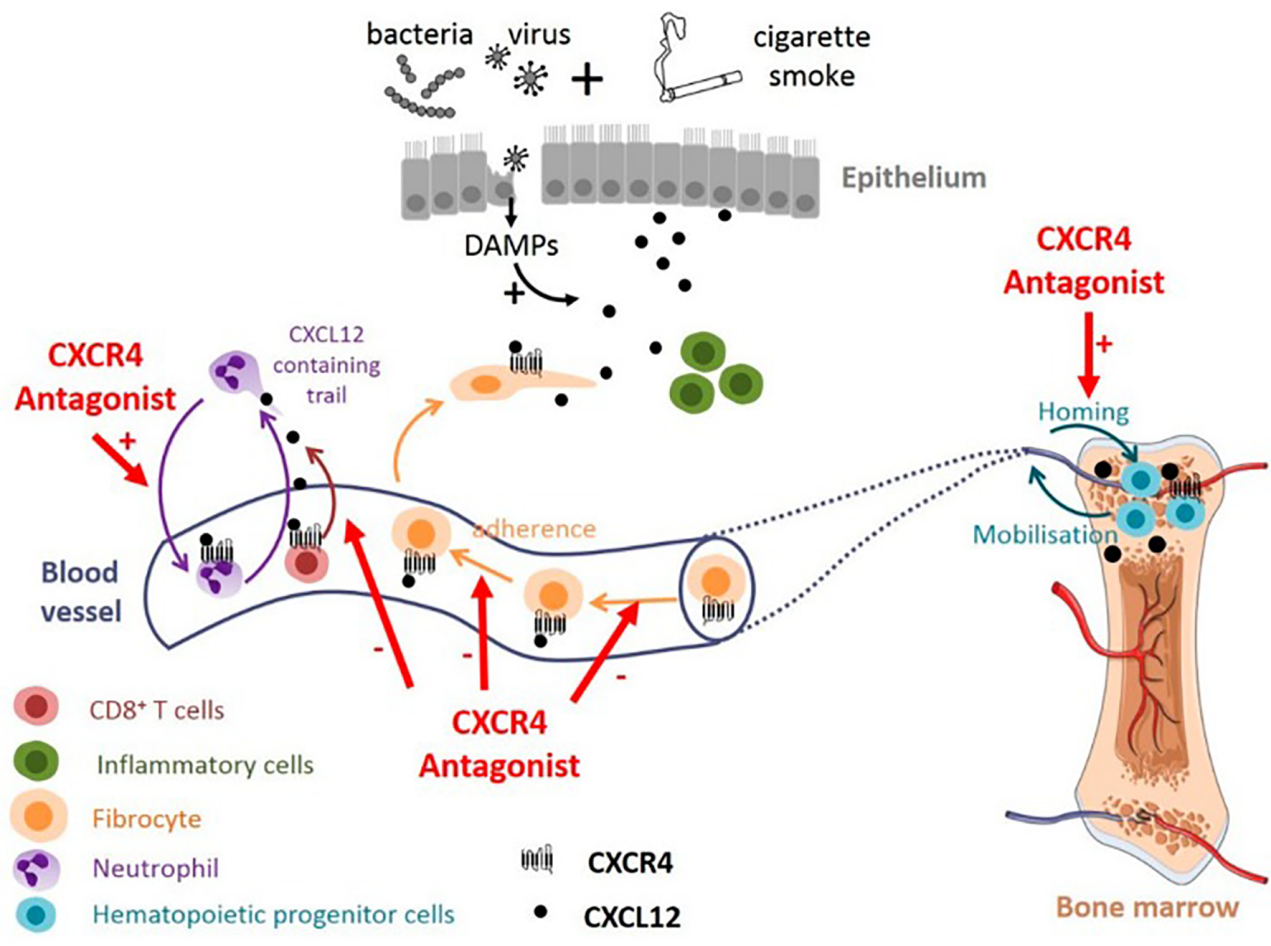
Potential effect of targeting CXCL12/CXCR4 axis in COPD. CXCR4 antagonists could promote neutrophil demargination from the lungs and inhibit T-cells and fibrocytes recruitment into bronchial tissue. CXCR4 antagonists may also contribute to maintaining the pool of hematopoeitic progenitor cells in the bone marrow, available for tissue repair (338).

All around the world, clinical trials are being conducted with the aim of targeting neutrophils in order to improve the conditions of patients with pulmonary infections and inflammations. Once a clear definition of the roles of neutrophils in pulmonary infections is established, researchers will be able to design effective treatments to target neutrophil dysregulation and development. Clinical trials of various promising pharmacological modulators of neutrophil recruitment/activation and NETosis are currently in Phase II and III. These include agents targeting NADPH oxidase, colony-stimulating factors and receptors, as well as CXCR2. In humans, CXCR2 appears to be the predominant neutrophil chemokine receptor despite having overlapping functions with CXCR1. CXCR2 has been implicated in several functions, including neutrophil egress from the bone marrow (331) and the activation of other cell types. CXCR1/CXCR2 have been a target for a number of pharmacological studies. For instance, patients with cystic fibrosis who were treated with a CXCR2 antagonist, SB-656933, had a decreased level of sputum inflammatory biomarkers (332). Another CXCR2 inhibitor, navarixin (MK-7123), improved pulmonary function in COPD, and the blockage of CXCR2 in patients suffering from moderate neutrophilic asthma, reduced the accumulation of neutrophil in the lungs (333). AZD5069, a selective CXCR2 antagonist, has been ineffective in controlling acute exacerbations in severe cases of refractory asthma, in spite of its ability to decrease neutrophil numbers in sputum (334) (**Table 4**). A phase II clinical trial of QBM076, another CXCR2 antagonist, was terminated due to elevated liver transaminase levels in patients with COPD (**Table 4**). In a murine experiment to inhibit CXCR2, an orally active CXCR2-antagonist molecule was used. It was confirmed that the molecule reduced inflammation caused by neutrophils, reduced infiltration of neutrophils to the lungs and decreased the level of enzymes that cause tissue damage (335). Danirixin, another CXCR2 antagonist, has also shown positive effects on respiratory symptoms and health status (336). Targeting the CXCL12-CXCR4 axis may also provide an effective treatment option for COPD. Plerixafor was found to reduce lung damage in mice with emphysema caused by cigarette smoke exposure (337). Although there are potential adverse effects in antagonizing the CXCL12-CXCR4 axis, it still provides a promising strategies in the treatment of pulmonary infections and inflammations (**Figure 6**). A summary of some selected clinical trials based on therapeutic strategies that specifically target neutrophils are provided in **Table 4**.

**Table 4:** Selected clinical trials targeting neutrophils in neutrophil-driven pulmonary inflammatory diseases.

Target	Name of drug	Company/sponsor	Indication	Phase	Identifier	Comments
CXCR2	AZD5069	AstraZeneca	Asthma	II	NCT01704495	Completed in 2014; the frequency of severe exacerbation in patients with severe asthma was not reduced
	QBM076	Novartis Pharmaceuticals	COPD	II	NCT01972776	Part 1 was completed; part 2 was terminated (for safety reasons) in 2015
	AZD5069	AstraZeneca	Asthma	I	NCT01890148	Completed in 2014. The inhibitor decreased sputum neutrophil numbers
NETs	Fostamatinib	NHLBI	COVID-19	II	NCT04579393	Ongoing
	Eculizumab	Hudson Medical	COVID-19	II	NCT04346797	Ongoing
	AIR DNase™	Protalix	Cystic fibrosis	II	NCT02722122	Status unknown
PDE4	Roflumilast	QuantumLeap Healthcare Collaborative	COVID-19	II	NCT04488081	Ongoing
NE	Alvelestat (AZD9668)	Mereo BioPharma	COPD	II	NCT03636347	Ongoing
	Lonodelestat (POL6014)	Santhera Pharmaceuticals	Cystic fibrosis	II	NCT03748199	Status unknown
	Elafin	Peking University Third Hospital	ARDS	I	NCT02944279	Completed in 2014
CXCR2	Danirixin (GSK1325756)	GlaxoSmithKline	COPD	II	NCT03034967	Completed in 2018
PDE4	Ensifentrine (RPL554)	Verona Pharma plc	COVID-19	II	NCT04527471	Ongoing
IL-6, IFNs, NETs	Baricitinib	Hospital of Prato	COVID-19	II/III	NCT04320277	Not yet recruiting

## Concluding Remarks

Neutrophils, which destroy pathogenic agents, have the ability to significantly modulate the functions of other immune cells and their recruitment into the lung. They play a major role in innate immunity during pulmonary inflammation. It is becoming increasingly clear that all of these processes are highly regulated by the signals they receive from their repertoire of PRRs which allow neutrophils, whose recruitment are modulated by chemokines, to sense PAMPs and DAMPs at the sites of inflammation. A holistic understanding of neutrophil pattern recognition pathways and the roles of CXC and CC chemokines in host immunity may allow for new approaches in the treatment of infectious and inflammatory disease of the lungs. In this review, we have demonstrated the roles and importance of PRRs, CXC and CC chemokines in host immunity during lung pathogenesis. We have herein summarize some of the signal transduction pathways through which neutrophils are recruited and guided to the lungs. The pathophysiological role of neutrophils in COVID-19 and thromboembolism have also been summarized. Finally, we discussed various neutrophilic biomarkers, and neutrophil-targeted therapies for neutrophil-driven pulmonary inflammatory diseases.

## Author Contributions

CYE conceptualized, structured and drafted the manuscript; EKD, CA, LD, SH, SL, SYA, EN, TS, XLZ, HL, ZX and FF contributed to literature search and writing of the manuscript. YW and XJZ contributed to the conceptualization, fund sourcing and supervision of the work. All authors contributed to the article and approved the submitted version.

## Funding

This work is supported by the National Natural Science Foundation of China (No. 81973099).

## Conflict of Interest

The authors declare that the research was conducted in the absence of any commercial or financial relationships that could be construed as a potential conflict of interest.

## Publisher’s Note

All claims expressed in this article are solely those of the authors and do not necessarily represent those of their affiliated organizations, or those of the publisher, the editors and the reviewers. Any product that may be evaluated in this article, or claim that may be made by its manufacturer, is not guaranteed or endorsed by the publisher.
